# A Splice Site Mutation in Laminin-α2 Results in a Severe Muscular Dystrophy and Growth Abnormalities in Zebrafish

**DOI:** 10.1371/journal.pone.0043794

**Published:** 2012-08-27

**Authors:** Vandana A. Gupta, Genri Kawahara, Jennifer A. Myers, Aye T. Chen, Thomas E. Hall, M. Chiara Manzini, Peter D. Currie, Yi Zhou, Leonard I. Zon, Louis M. Kunkel, Alan H. Beggs

**Affiliations:** 1 Genomics Program and Division of Genetics, Boston Children’s Hospital, Harvard Medical School, The Manton Center for Orphan Disease Research, Boston, Massachusetts, United States of America; 2 Stem Cell Program and Pediatric Hematology/Oncology, Boston Children’s Hospital and Dana Farber Cancer Institute, Harvard Stem Cell Institute, Harvard Medical School, Boston, Massachusetts, United States of America; 3 Australian Regenerative Medicine Institute, Monash University, Clayton Campus, Victoria, Australia; 4 Howard Hughes Medical Institute, San Francisco, California, United States of America; Johns Hopkins Univ. School of Medicine, United States of America

## Abstract

Congenital muscular dystrophy (CMD) is a clinically and genetically heterogeneous group of inherited muscle disorders. In patients, muscle weakness is usually present at or shortly after birth and is progressive in nature. Merosin deficient congenital muscular dystrophy (MDC1A) is a form of CMD caused by a defect in the laminin-α2 gene (*LAMA2*). Laminin-α2 is an extracellular matrix protein that interacts with the dystrophin-dystroglycan (DGC) complex in membranes providing stability to muscle fibers. In an N-ethyl-N-nitrosourea mutagenesis screen to develop zebrafish models of neuromuscular diseases, we identified a mutant fish that exhibits severe muscular dystrophy early in development. Genetic mapping identified a splice site mutation in the *lama2* gene. This splice site is highly conserved in humans and this mutation results in mis-splicing of RNA and a loss of protein function. Homozygous *lama2* mutant zebrafish, designated *lama2^cl501/cl501^*, exhibited reduced motor function and progressive degeneration of skeletal muscles and died at 8–15 days post fertilization. The skeletal muscles exhibited damaged myosepta and detachment of myofibers in the affected fish. Laminin-α2 deficiency also resulted in growth defects in the brain and eye of the mutant fish. This laminin-α2 deficient mutant fish represents a novel disease model to develop therapies for modulating splicing defects in congenital muscular dystrophies and to restore the muscle function in human patients with CMD.

## Introduction

Congenital muscular dystrophies (CMDs) are a clinically and genetically heterogeneous group of neuromuscular disorders that typically present at birth or in early infancy with hypotonia, muscle weakness, and histological evidence of a dystrophic myopathy [1], [2], [3], [4]. MDC1A is a specific form of CMD associated with absence or reduction of laminin-α2 in skeletal muscle [5], [6]. MDC1A, which represents approximately 50% of CMD cases, is caused by recessive mutations in the *LAMA2* gene encoding the α2 chain of laminin [6]. More than 800 mutations in the *LAMA2* gene have been identified to date in both coding as well as non-coding sequences (www.dmd.nl). In addition to the skeletal muscle defects, the brains of MDC1A patients exhibit white matter abnormalities that appear in many patients after 2 years of age. Epilepsy and focal cortical dysplasia leading to cognitive deterioration have also been seen in patients affected with MDC1A [7], [8], [9]. Patients with *LAMA2* mutations exhibit feeding problems and/or respiratory difficulties and often require feeding tube placement or ventilator assistance in severe cases [10].

Laminins are a family of large extracellular glycoproteins. Laminin-α2 is specifically expressed in the basal lamina of striated muscles and peripheral nerves. Laminin-α2 interacts with laminins β1, γ1 or β2 to form laminin 211 and 221 heteromers in the basal lamina [11], [12]. These laminin complexes interact with α-dystroglycan at the surface of myofibers, resulting in stabilization of the sarcolemma as mutations abolishing these interactions are associated with inability of muscles fibers to adhere to the basement membrane [13]. Laminins also play a significant role in muscle repair as laminin-α2 deficient muscles exhibit a reduction in proliferative ability of satellite cells and inability to regenerate [14], [15]. A number of animal models of laminin-α2 deficiencies have been developed. In mice, a complete loss of laminin-α2 results in severe muscular dystrophy and growth retardation while missense or splice-site mutations resulting in truncated Lama2 protein in muscle cause a less severe phenotype [16], [17], [18]. Laminin-α2 deficiency is also associated with muscular dystrophy and brain defects in cats [19]. Similar clinical and pathological changes in animal models as seen in human patients suggest an evolutionary conservation of laminin-α2 function in vertebrate muscles [20].

MDC1A is associated with severe disability and shorter lifespan and no specific therapies are available to treat this disease. While targeted therapeutic developments using murine models appear to be promising, testing a large number of drugs in mice can be very slow, signifying the need to develop animal models that can be used for high throughput therapeutic screens. In the past decade, zebrafish have emerged as a powerful genetic model to study diseases and to develop therapies due to high genomic similarity with humans, small size, fast reproductive rates and ease of doing high throughput drug screens [21], [22], [23], [24]. Therefore, we performed an N-ethyl-N-nitrosourea (ENU) mutagenesis screen in zebrafish to develop vertebrate animal models of neuromuscular diseases to enable the appraisal of early pathological processes and subsequent trial of therapeutic approaches. This study describes the genetic mapping and characterization of a mutant zebrafish line with a novel splice site mutation in the *lama2* gene causing muscular dystrophy and growth defects. These mutant fish model the disease pathology associated with human CMD patients and will be a novel disease model to test therapies based on splicing-modulation as well as performing high throughput chemical screens.

## Results

### A Severe Muscular Dystrophy in Zebrafish Results Due to a Splice Site Mutation in *Lama2* Gene

**Figure 1:**
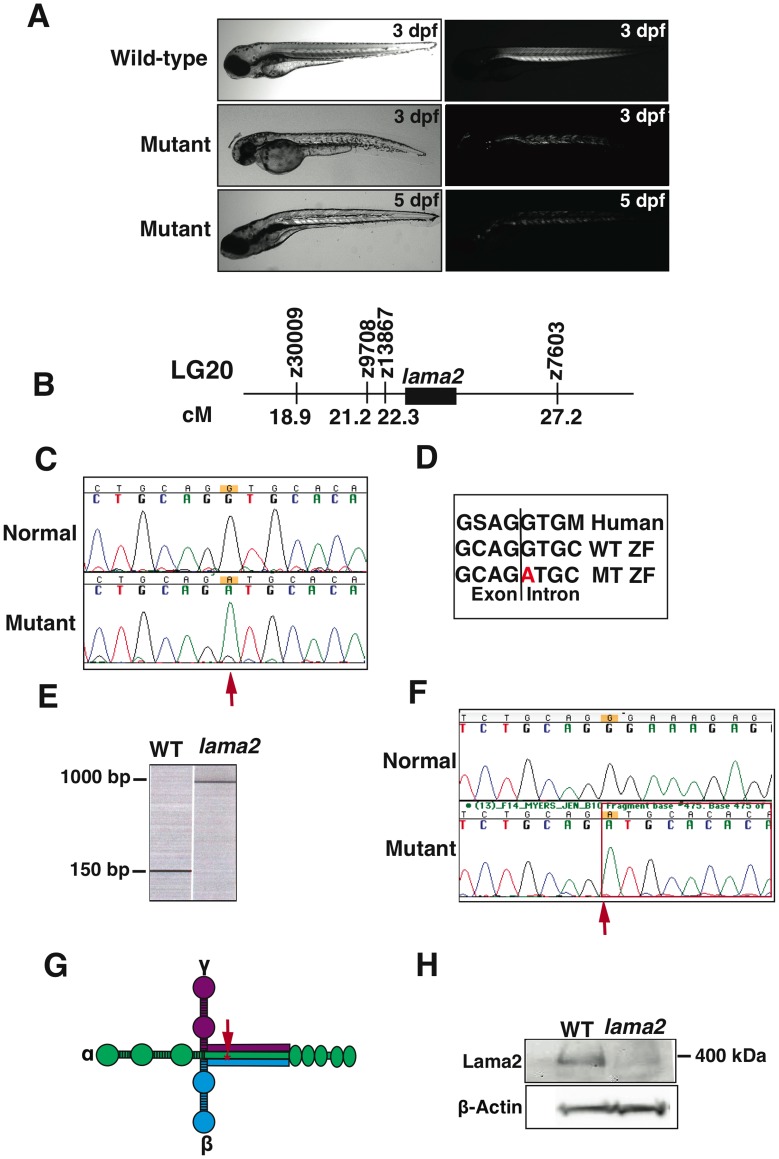
Morphological, genetic and molecular characterization of *lama2^cl501/cl501^* zebrafish. (A) In comparison to the wild-type fish, *lama2^cl501/cl501^* fish exhibit smaller bodies (left panel) and displayed muscle disorganization early in development at 3 dpf (right panel). The muscle degeneration progressed to most of the somites by 5 dpf. (B) Genetic mapping in mutant fish associated the linkage of mutant locus in 4.9 cM region on chromosome 20 in zebrafish identifying *lama2* as the candidate gene. (C) Sequencing of *lama2* gene in zebrafish identified a mutation at c.5376+1G>A in the mutant fish (arrow). (D) The consensus splice site containing the mutation is highly conserved in human *LAMA2* gene. (E) RT-PCR using primers specific for the exons 33 and 34 on either side of the mutant intronic sequence showed a larger PCR product (∼900 base pairs) in the mutant fish in comparison to the wild-type fish (152 base pairs) suggesting aberrant splicing. (F) Sequencing of the RT-PCR products revealed the retention of 764 base pairs of intron 33 in the mutation fish (arrow). (G) This mutation is localized at the long arm coiled-coil of lama2 protein (arrow) that interacts with β- and γ- laminins to form functional laminin complex (H) Western blotting analysis showed a reduction of laminin-α2 protein in the mutant fish.

A forward genetic screen to identify genes involved in neuromuscular disorders using zebrafish resulted in the identification of a mutant that exhibited severe muscular dystrophy. Wild-type embryos displayed highly birefringent skeletal muscles indicative of organized sarcomeric structure within muscle fibers. The mutant fish, however, displayed patchy birefringence suggesting highly degenerative skeletal muscles evident by 3 days post fertilization (dpf) (Fig. 1A). This dystrophic phenotype was much more severe than seen in dystrophin or dystroglycan null zebrafish mutants where extensive muscle degeneration becomes prominent later in development (4–5 dpf) [13], [25]. The muscle degeneration progressed to most of the somites by 5 dpf (Fig. 1A). The mutant fish exhibit reduced mobility in comparison to the wild-type fish. In an embryo swirl assay, swirling of petri dish containing embryos results in collection of embryos in the middle of the dish due to forces around the edge of the dish. As the swirling motion stops, embryos exhibit a swimming response towards the periphery of the dish. After cessation of swirling, the total number of wild-type and mutant embryos that remained in the middle of the dish (3 cm diameter) was counted. While, most of the wild-type embryos swam out of the middle circle immediately (82.89% escaped, n = 110), a large number of mutant embryos failed to swim out of the circle (41.94% escaped, n = 38) demonstrating reduced motility. The motor function of muscle was also assessed using touch evoked response assay. Mutant fry invariably exhibited slower swimming behavior than their wild type clutch-mates in touch-evoke response assay (wild-type: 4.8±1.1 cm/0.1 s, n = 6; mutant: 1.2±0.6 cm/0.1 s, n = 6; Videos S1 and S2). These data demonstrate that mutation in these fish results in impaired motor function. Heterozygous fish appeared unaffected and had normal lifespans and fecundity while homozygous mutant fish invariably died between 8–15 dpf, much before they attain reproductive age.

To identify the mutant locus, genetic mapping of the dystrophic phenotype was performed using SSLP markers. An initial bulk segregant analysis mapped the putative gene to chromosome 20 between z13867 (22.3 cM) and z7603 (27.2 cM). An analysis of this genomic area (4.9 cM) identified *lama2* as a potential candidate gene (Fig. 1B). Therefore genetic complementation analysis was performed by crossing the mutant fish line with a previously known fish mutant of *lama2^tk209^* (*candyfloss)* [20]. These mutants failed to genetically complement each other suggesting a mutation in the same gene. To identify the mutation in *lama2* gene (herein designated *lama2^cl501^*), genomic sequencing was performed that identified a point mutation in c.5376+1G>A at a donor splice site in intron 33 (Fig. 1C). This G is 100% conserved in all vertebra examined and any base change at this position results in aberrant splicing of mRNA [26]. This splice site further showed high homology to the consensus sequence in the human *LAMA2* gene (Fig. 1D).

To understand the effect of this mutation on splicing, RT-PCR analysis was performed in wild-type and mutant fish. RT-PCR using primers specific for exons 33 and 34 showed the presence of a larger PCR product in the mutant fish in comparison to wild-type fish (Fig. 1E). Sequencing of this PCR product from *lama2^cl501/cl501^* fish identified an insertion of 764 base pairs of intron 33 in the mutant fish (Fig. 1F). Insertion of this intronic sequence resulted in a frame-shift mutation and generation of stop codon in the mutant laminin-α2 mRNA corresponding to the long arm coiled-coil in the protein that is required to interact with β- and γ- laminins to form the laminin complex in basal lamina (Fig. 1G, arrow). Western blotting using a laminin-α2 antibody identified an expected size high molecular weight protein band (∼400kDa) in wild-type fish, which was reduced in the mutant fish (∼90%) (Fig. 1H). This suggests that the splice site mutation in the *lama2* gene resulted in a loss of protein function in mutant zebrafish.

### *Lama2^cl501/cl501^* Zebrafish Exhibit Damaged Myosepta and Detached Myofibers in Muscles

**Figure 2:**
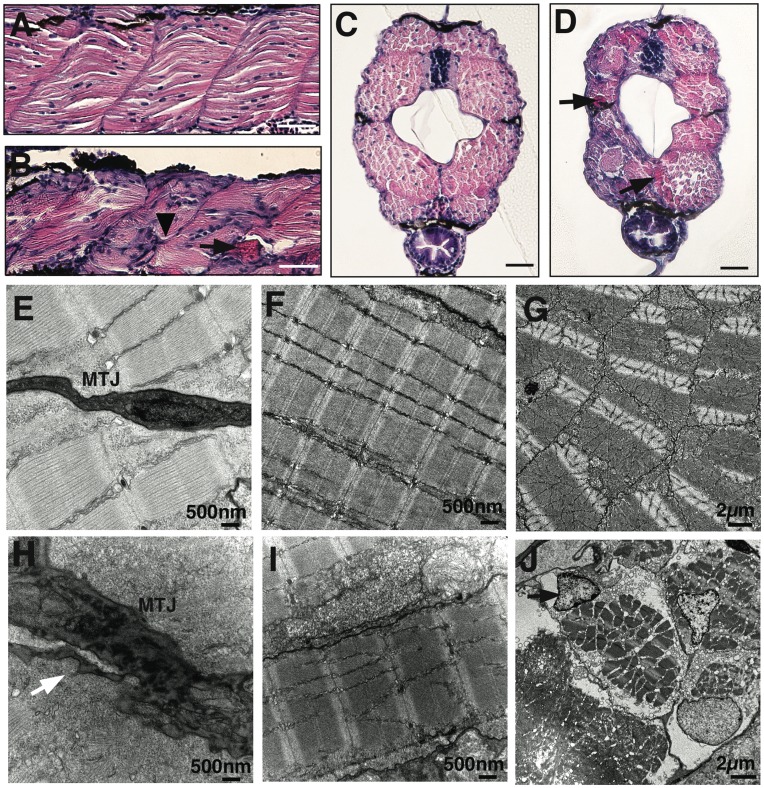
Abnormal myosepta and myofiber detachment in laminin-α2 deficient *lama2^cl501/cl501^* fish. (A & B) Hematoxylin and eosin staining of longitudinal sections of wild-type and *lama2* mutant fish at 5 dpf. Mutant muscles showed highly disorganized myofibers in the affected somites with irregular myosepta boundaries (arrowhead) and eosin positive detached myofibers (arrow). (C–D) Cross-sections of wild-type and mutation fish also showed smaller myotome and degenerating muscle fibers in *lama2* mutant fish at 5 dpf (arrows), bars  = 10 µm. (E & H) Electron microscopy showed myofiber detachment from the myotendinous junction (MTJ) in mutant muscles (arrow). (F & I) The myofibers in wild-type muscles attached tightly to the surrounding fibers while mutant muscle displayed large gaps in the extracellular matrix between adjacent fibers and disorganized Z- lines (black arrow) and M-lines (while arrow). (G & J) Defects in extracellular-matrix results in damaged myofibers in the myotome (cross-section). A large number of apoptotic nuclei were observed in the mutant muscles (J, arrow).

To identify the pathological changes in the skeletal muscles of *lama2^cl501/cl501^* mutant fish, histological analysis was performed by hematoxylin and eosin staining at 5 dpf. Analysis of longitudinal sections identified multiple abnormalities in the skeletal muscles of the mutant fish (Fig. 2A–B). *Lama2^cl501/cl501^* fish displayed muscular atrophy with disrupted myosepta in most of their somites (Fig. 2B, arrowhead). In addition, several detached myofibers were seen to be present in the mutant fish (Fig. 2B, arrow). Cross-sections of wild-type and mutant fish revealed smaller myotomes in the mutant fish in comparison to the wild-type fish (Fig. 2C–D). Cross-sections of the mutant muscles also showed eosin positive hypercontracted damaged myofibers indicative of dystrophic muscles (Fig. 2D, arrows).

### Laminin-α2 Deficient Zebrafish have Extracellular Matrix Defects in the Skeletal Muscles

To identify the ultra-structural changes in skeletal muscles due to laminin-α2 deficiency, electron microscopy was performed in the control and *lama2^cl501/cl501^* fish. In wild-type fish, laminin is expressed in the extra-cellular matrix at the myotendinous junction (MTJ). Components of the dystrophin-DGC complex are expressed at the end of myofibers that attach to the MTJ during early zebrafish development. The myotome of wild-type muscles exhibited well -organized extra cellular matrix (Fig. 2E). Mutant fish exhibited highly damaged MTJ with thickening of extracellular areas and accumulation of electron dense collagen fibers and branching projections. (Fig. 2H, arrow). The myofibers that remained attached to the myosepta further showed disorganized Z- and M-lines in the contractile apparatus without any sarcolemmal defects (Fig. 2F and 2I). Cross-sectional views of the muscle revealed detachment of myofibers from the surrounding basement membrane and accumulation of abnormal membrane vesicles that are absent in wild-type muscles, as well as apoptotic nuclei (Fig. 2G and 2J, arrow), suggesting the presence of necrotic fibers.

**Figure 3:**
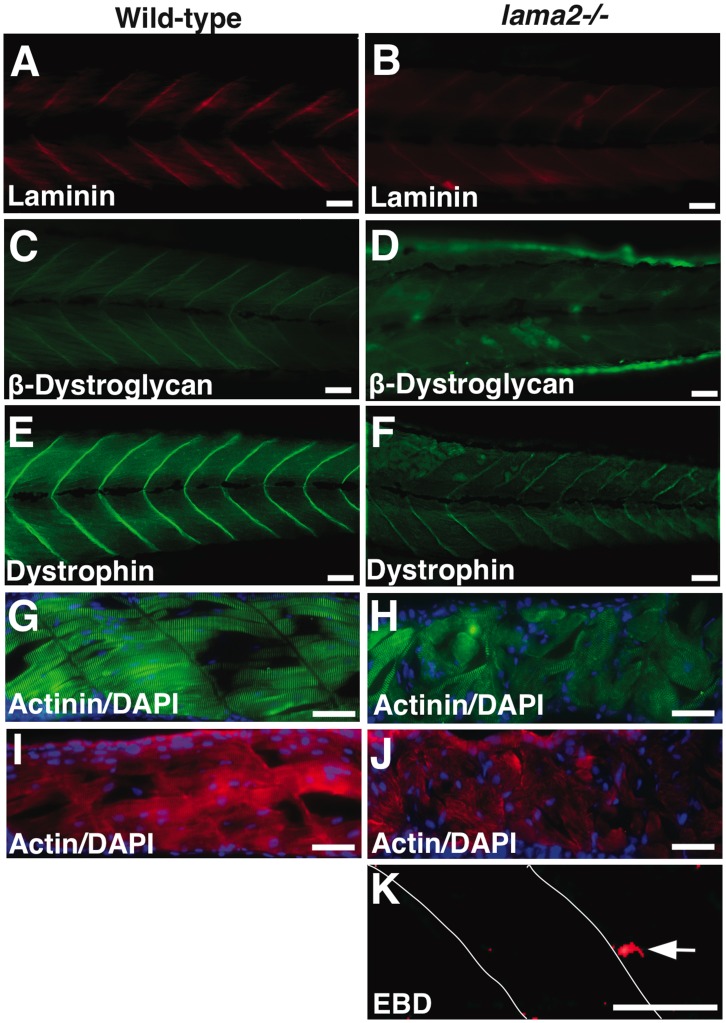
Laminin-α2 deficiency results in severe muscle degeneration. (A–B) Wholemount immunofluorescence analysis showed reduced levels of laminin complex in myotendinous junctions in the mutant muscles. (C–F) The expression of β-dystroglycan as well as dystrophin was also reduced in the mutant muscles. (G–J) Myofibers in wild-type muscles are attached to either side of the myotendinous junctions and displayed well-organized muscles. Mutant fish, however, displayed highly disorganized muscles. Several detached myofibers lacking the contractile proteins are seen in the mutant muscles by α-actinin and sarcomeric actin antibody staining (arrows). (K) Evans blue dye (EBD) injections at 3 dpf detected occasional staining in necrotic fibers (arrow). Bars  = 10 µm.

To understand the molecular effect of laminin-α2 deficiency on muscle, whole mount immunofluorescence was performed at 5 dpf. Immunofluorescence using a pan anti-laminin antibody showed that the overall expression of laminins was reduced in the mutant muscles. This implies that loss of laminin-α2 destabilizes the laminin complex in basal lamina in zebrafish (Fig. 3A–B). The expression of membrane β-dystroglycan and dystrophin was also reduced in the mutant fish (Fig. 3C–F). Immunofluorescence in a large number of embryos revealed a direct correlation between muscle degeneration and reduction of sarcolemmal proteins and highly reduced levels of sarcolemmal proteins were seen in more severely affected embryos. Immunofluorescence staining for α-actinin and actin also detected highly disorganized muscles with hypercontracted myofibers lacking myofibrillar organization in the muscles of the mutant fish, indicative of severe muscle degeneration in the skeletal muscle (Fig. 3G–J). To examine if the sarcolemmal damage is a direct result of lama2 deficiency or is a secondary consequence of extensive fiber necrosis, Evans blue dye (EBD) injections were performed in live zebrafish embryos during early development stage at 3 dpf. Analysis of mutant somites failed to detect any EBD positive intact myofibers in the mutant fish except the occasional dye signal from the necrotic fibers (Figure 3K). This suggests that sarcolemmal damage observed in *lama2* mutant fish is due to extensive muscle necrosis during advanced stages of disease progression.

### *Lama2* Mutant Fish Exhibit Growth Abnormalities

**Figure 4:**
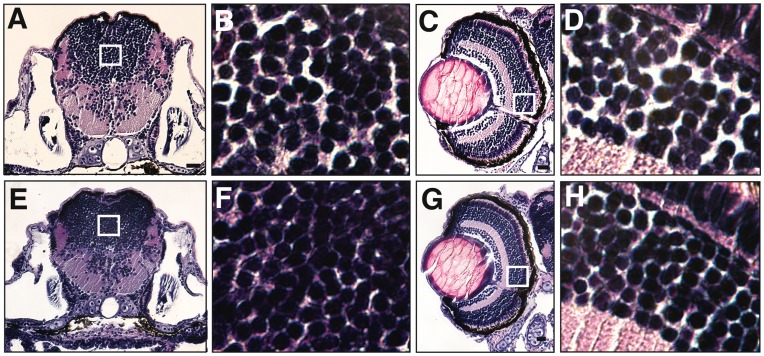
*Lama2^cl501/cl501^* fish exhibit growth abnormalities. (A & E) Histological analysis of cross-section at 7 dpf revealed smaller brain in the mutant fish in comparison to wild-type fish. (B & F) Magnified views of brains showing tightly clumped cells in the mutant brains. (C & G) Wild-type as well as mutant fish displayed well-organized cellular layers in eyes. (D & H) Magnified views of the ganglion cell layer showed tightly organized cells with reduced extracellular space between the cellular layers in mutant fish in comparison to the wild-type.

Laminin-α2 deficiency in humans is associated with brain abnormalities. Therefore, to identify any brain defects associated with laminin-α2 deficiency in zebrafish, histological analysis of the *lama2^cl501/cl501^* CNS was performed using hematoxylin and eosin staining (Fig. 4A–B and 4E–F). A comparison of brain histology in several embryos showed a consistent reduction in the size of brain in the mutant fish (11±5.81%) at 7 dpf. The cells in the mutant brain appeared to be clumped in comparison to the wild-type fish. Further, a comparison of eyes in wild-type and mutant fish also consistently revealed smaller eyes (16±7.83%) in the mutant fish with compressed cellular layers (Fig. 4C–D and 4G–H). Similar to the brain, cells in the ganglion layer in the eye appeared to be tightly clumped in the mutant fish as compared to the wild-type. This is consistent with the previously published reports of growth retardation in mice in laminin-α2 deficiency, suggesting that similar processes may be altered in zebrafish [16].

## Discussion

Congenital muscular dystrophies represent a severe group of muscular dystrophy for which no treatment is available. Here we describe a novel splice site mutant of laminin-α2 deficiency modeling congenital muscular dystrophies as seen in human patients. Of more than 700 pathogenic mutations reported in the human *LAMA2* gene, 30–40% of mutations are present within splice sites (www.dmd.nl). We show here that a novel splice-site mutation in *lama2* in zebrafish results in muscular dystrophy as well as brain and eye abnormalities. There are several animal models of laminin-α2 available that exhibit mild to severe form of dystrophy depending on the nature of mutation, including dy2J mice that have a splice-site mutation [16], [18], [20]. However, the consequences of splice site mutation in *lama2^cl501/cl501^*are different from those in the dy2J mice and are in a distinct species/model system. In dy2J mice, another alternative splice site is used by the splicing machinery causing the expression of a smaller protein lacking 55 amino-acids in the domain IV. Expression of this smaller laminin-α2 protein results in a milder form of dystrophy in dy2J mice as compared to dy3k mice that have a null mutation. The splice site mutation of *lama2^cl501/cl501^* zebrafish results in an intron retention and a corresponding frameshift mutation leading to generation of a stop codon and a complete lack of laminin-α2 protein, resembling a null mutation. Both of these splice site mutants result in different degrees of lama2 expression (partial vs. null), as well as disease severity, representing distinct disease models. It has also been shown that *candyfloss* fish has a nonsense mutation in the C-terminal domain of Lama2 resulting in dystrophic muscles [20]. While the disease pathology of *lama2^cl501/cl501^* is similar to *candyfloss* mutant, two mutations are different at the molecular levels.

In human MDC1A, a phenotype-genotype correlation is often seen as partial deficiency of protein leads to a milder form of the disease and complete loss of function results in severe muscle and brain abnormalities [27]. In recent years, large efforts have been made to develop therapies based on splice-modulation. A successful application of this approach is seen in Duchenne muscular dystrophy where skipping of mutant exon by antisense oligonucleotides results in restoration of open reading frame and a smaller functional protein with less severe Becker muscular dystrophy. This approach is currently in clinical trials for exon 51 skipping in Duchenne muscular dystrophy [28], [29]. In several other disorders, splice modulation by restoring cryptic splicing, tweaking alternative splicing or inducing exon inclusion has already been shown to have great potential for treating diseases [30], [31]. Studies have also identified several classes of chemicals that can correct the splicing defects and will be attractive to test in our zebrafish models for future studies [32]. Therefore, *lama2^cl501/cl501^* zebrafish model will be a valuable tool in testing therapies aimed in correcting the muscle defects and decreasing disease severity in patients affected with splice-site mutations in *LAMA2* gene. The small size, rapid ex-vivo development, and live analysis of zebrafish embryos during earlier stages in development makes evaluating these therapies much faster than it would be using higher vertebrate animal models. The *lama2^cl501/cl501^* fish have a severe and easily assayable phenotype that will provide robust endpoints, such as birefringence, reduced motility and increased mortality, for testing potential therapies. In addition, therapies aimed at restoring the muscle function in mutant fish by modulating pathways directly or indirectly regulating laminin-α2 function and/or stabilizing the detaching myofibers from extracellular matrix, will potentially be applicable in treating human patients affected with MDC1A. Transgenic as well as protein therapy studies in mice have shown a great potential for treatment of MDC1A by upregulation of Lama1 or integrins in the muscle [33], [34]. Therefore, identification of chemicals that can upregulate these pathways will be helpful for treating patients with Lama2 deficiency. Further these therapies will be tested in *candyfloss* mutants as well as different mouse models for their robustness in muscle improvement.

The *lama2^cl501/cl501^* mutant fish showed similar pathological defects in the extra-cellular matrix and extensive myofiber damage to those seen in human patients. Similar to the neonatal onset of the disease in human patients, *lama2^cl501/cl501^* fish also exhibited muscle degeneration early in development (3 dpf). Moreover, no regenerative muscles fibers were detected in the skeletal muscles of the affected fish. No sarcolemmal defects were seen during earlier stages of disease pathogenesis as seen by a lack of EBD staining which is similar to a previously published study in *candyfloss* fish that showed extracellular matrix defects are not associated with sarcolemmal damages in zebrafish. Although it is not practical to perform EBD injections at later ages, ultrastructural and immunohistochemical studies during later stages revealed development of extensive degeneration of skeletal muscles in *lama2^cl501/cl501^* fish with a corresponding reduction in β-dystroglycan and dystrophin expression. Hayashi et al., have shown that in a severe congenital form of MDC1A, extensive muscle degeneration during neonatal phases results in activation of membrane attack complex (MCA) that immunolocalized to myofibers showing highly reduced levels of β-dystroglycan and dystrophin suggesting that degenerating myofibers start to lose the immunoreactivity of sarcolemmal proteins [35]. Similarly, another study reported a patient exhibiting deficiency of Lama2 also exhibited low levels of dystrophin, suggesting sarcolemmal damage may be secondary to ECM defects that manifest in highly degenerative and necrotic fibers [36]. These observations suggest that, similar to human patients, early dystrophic changes in fish involve extra-cellular matrix defects and fiber detachment without significant damage to the sarcolemma. With disease progression, extensive myofiber damage and necrosis results in a reduction of sarcolemmal proteins in the affected muscles in zebrafish as has been seen in severe congenital forms of the disease in humans [35]. This mode of muscle damage is quite distinct from *sapje* fish harboring a mutation in dystrophin where sarcolemmal damage is evident during early phases of disease progression [25].

The laminin-α2 deficient fish also exhibited a reduction in body size as seen in the muscle, brain and eyes. Previously, it has been shown that complete knockout of *Lama2* in mice resulted in a generalized growth retardation and severe muscular dystrophy and death by 5 weeks of age [16] suggesting similar pathological processes being altered in all vertebrate models. In the murine model, laminin deficiency has been shown to cause delayed oligodendrocyte maturation and contribute to the CNS defects seen in MDC1A [37]. However, no significant structural abnormalities were observed in the brains of laminin-α2 deficient fish by 5 dpf considering strong expression of *lama2* transcripts is seen in muscle, eye and brain of developing zebrafish embryos [38]. In MDC1A, the white matter defects are normally seen after six months or later in human patients. A lack of detection of similar changes in the zebrafish brain could be due to early lethality of *lama2* mutations before such processes become apparent in zebrafish and require future investigation.

As *lama2^cl501/cl501^* mutant fish recapitulate the pathological findings and muscle defects seen in MDC1A, we propose this novel splice site zebrafish mutant will be an invaluable vertebrate animal model for developing therapies and improving muscle function in the congenital muscular dystrophies.

## Materials and Methods

### Fish Maintenance

Fish were bred and maintained using standard methods as described [39]. Wild-type embryos were obtained from the Oregon AB line and were staged by days post fertilization (dpf) at 28.5°C. All animal work was performed with approval from the Children’s Hospital Boston Animal Care and Use Committee (11-05-1955R).

### ENU Screen

The ENU mutagenesis screen was performed as described earlier [13]. Zebrafish larvae were screened at 3–4 dpf using a birefringence assay. Crosses in which approximately 25%±5% of larvae showed patchy or reduced birefringence of their axial skeletal muscles in polarized light were identified as potential skeletal muscle mutants and these families were selected for further study (n = 70–200).

### Motility Assay

Embryo swirl assay was performed by swirling the petri dishes containing wild-type and mutant embryos till all the embryos collected in the middle of the dish. As the water whorl is stopped, total number of embryos left in the middle of the dish (3 cm diameter) was counted (n = 60–150).

For touch evoke response assay, mechanosensory stimuli were delivered to 3 dpf embryo tails using insect pins. Time-lapse images of zebrafish embryos were takes at different time intervals using a Nikon smz1500 microscope with SPOT camera system. The length of time for each fish to leave the frame of view was averaged across fish (n = 6) [13].

### Genetic Mapping

Mutant heterozygous zebrafish (AB strain) were out-crossed to wild-type *wik* to generate polymorphic mapping strains. Low-resolution mapping was done with 40 diploid mutant and 40 diploid wild-type embryos obtained from in-crossing mapping F_2_ fish. Microsatellite CA markers throughout the genome were used to scan for linkage as described [40]. Complementation analysis was performed to determine if this mutant fish and *lama2^tk209^* (*candyfloss*) were allelic by crossing heterozygous fish. Mutant embryos were evaluated by birefringence assay. Genomic sequencing was performed in wild-type and mutant fish using primers spanning the complete *lama2* gene in zebrafish (Pubmed accession Number: JN786913.1). As the sequence of zebrafish *lama2* gene was already known, no new sequence was generated. Therefore, no sequence was submitted to GenBank.

### RNA Extraction and RT-PCR

RNA was isolated from pools of (3–5) mutant or wild-type embryos at 5 dpf using RNeasy fibrous tissue mini kit (Qiagen). cDNA was prepared using Superscript III first-strand synthesis kit (Invitrogen). Equal concentrations of wild-type and mutant RNA were used for cDNA synthesis. RT-PCR was performed using exonic primers and PCR products were analyzed in using the HAD-GT12 Genetic Analyzer (Qiagen).

### Histology, Immunofluorescence

Fish embryos and larvae were anesthetized and fixed overnight in 4% paraformaldehyde in PBS at 4°C. For histology, 5 um thin paraffin sections were cut stained with hematoxylin and eosin (Rodent Histopathology Core, Harvard Medical School). Immunofluorescence was performed using the protocol described previously [13]. Whole mount immunofluorescence was performed on wild-type and mutant embryos (15–20) as described [13].

Primary antibodies used in this study were: β-dystroglycan (Novocastra, NCL-b-DG), laminin-α2 (Sigma, HPA005537), dystrophin (Sigma, D8043), laminin (Sigma, L9393), α-actinin; clone EA-53 (Sigma, A7811). Nuclear staining was done using DAPI. Secondary antibodies were purchased from Jackson ImmunoResearch.

### Electron Microscopy

Zebrafish embryos (n = 8–10) were fixed in formaldehyde-glutaraldehyde-picric acid in cacodylate buffer overnight at 4°C followed by osmication and uranyl acetate staining. Subsequently, embryos were dehydrated in a series of ethanol washes and finally embedded in Taab epon (Marivac Ltd., Nova Scotia, Canada). 95 nm sections were cut with a Leica ultracut microtome, picked up on 100 m formvar coated Cu grids and stained with 0.2% lead citrate. Sections from 3–4 embryos were viewed and imaged under the Philips Tecnai BioTwin Spirit Electron Microscope (Electron Microscopy Core, Harvard Medical School).

## Supporting Information

Video S1 **Touch evoke response assay in wild-type embryos at 5 dpf.**
(MP4)

Video S2 **Touch evoke response assay in** ***lama2*** **mutant fish at 5 dpf.**
(MP4)
